# Bilateral Serous Retinal Detachment Associated With Nonhypertensive Preeclampsia: A Case Report

**DOI:** 10.7759/cureus.104306

**Published:** 2026-02-26

**Authors:** Özge Oztürk, Sina Hakami

**Affiliations:** 1 Ophthalmology Department, Erasmus Hospital, Brussels, BEL; 2 Ophthalmology Department, Université Libre de Bruxelles, Brussels, BEL

**Keywords:** case report, ophthalmology, pre-eclampsia, pregnancy, serous retinal detachment

## Abstract

Preeclampsia is a pregnancy-specific multisystem disorder characterized by new-onset hypertension and proteinuria, which may present with a variety of other maternal organ dysfunctions after 20 weeks of gestation. Although ocular complications are uncommon, serous (exudative) retinal detachment (SRD) can occur, especially in association with severe preeclampsia or HELLP (hemolysis, elevated liver enzymes, low platelets) syndrome. While SRD may cause some discomfort for the patient due to decreased visual acuity resulting from its central location, full recovery is the rule, within a maximum of 12 weeks.

Here, we report a case of bilateral SRD in a 27-year-old nulliparous woman at 32 weeks’ gestation, with clinical features of preeclampsia despite only modest blood pressure elevation. After cesarean delivery at 32+3 weeks for preeclampsia with severe features, the patient had full anatomical and functional recovery by 1 month, with stable findings at 3 months.

This case highlights that SRD may complicate preeclampsia even when absolute blood pressure values are not markedly elevated and underscores the importance of prompt ophthalmic assessment in pregnant patients with visual complaints.

## Introduction

Preeclampsia is a pregnancy-specific multisystemic disorder that affects approximately 2-8% of pregnancies and is classically characterized by new‑onset hypertension (≥140/90 mmHg) after 20 weeks of gestation, together with proteinuria or other signs of maternal organ dysfunction [1,2]. Preeclampsia encompasses a range of clinical presentations, including severe preeclampsia (blood pressure ≥160/110 mmHg or evidence of end‑organ damage), eclampsia, and HELLP (hemolysis, elevated liver enzymes, low platelets) syndrome [3]. Eclampsia refers to seizures that occur in a woman with preeclampsia, which are not caused by any other condition, while HELLP syndrome is characterized by hemolysis, elevated liver enzymes, and low platelets [3].

Different ophthalmic conditions have been associated with preeclampsia, such as cortical blindness, serous retinal detachment (SRD), Purtscher-like retinopathy, central retinal vein occlusions, and retinal or vitreous hemorrhages [1]. SRD is a rare complication of preeclampsia (reported in roughly 0.1-2% of cases) that may appear before, during, or after delivery and can be unilateral or bilateral. It is defined as a separation of the neurosensory retina from the underlying retinal pigment epithelium and manifests as visual loss, usually without photopsia or floaters, the latter being more specific to a rhegmatogenous retinal detachment [2].

Although the majority of reported cases occur in patients with arterial hypertension [3], this case report demonstrates that SRD may occur in the absence of hypertension and underscores the importance of a comprehensive ophthalmologic evaluation in patients presenting with visual symptoms, even when systemic parameters, including blood pressure, are within normal limits.

## Case presentation

A 27-year-old nulliparous woman, at 31+4 weeks of gestation, was referred to the gynecology department for proteinuria (3+) with borderline elevated blood pressure. Upon admission, she had peripheral edema and a blood pressure of 138/94 mmHg. Laboratory tests showed a serum creatinine level of 1.3 mg/dL. Biological tests performed throughout the hospital stay showed normal liver function, with the highest ALT and AST levels being 13 IU/L and 10 IU/L, respectively. The lowest platelet (PLT) count was 155,000 PLT. The remainder of the clinical examination of the patient was unremarkable. Fetal assessment revealed stage I intrauterine growth restriction (IUGR) but was otherwise normal. Based on these findings, betamethasone was administered for fetal lung maturation. 

**Figure 1:**
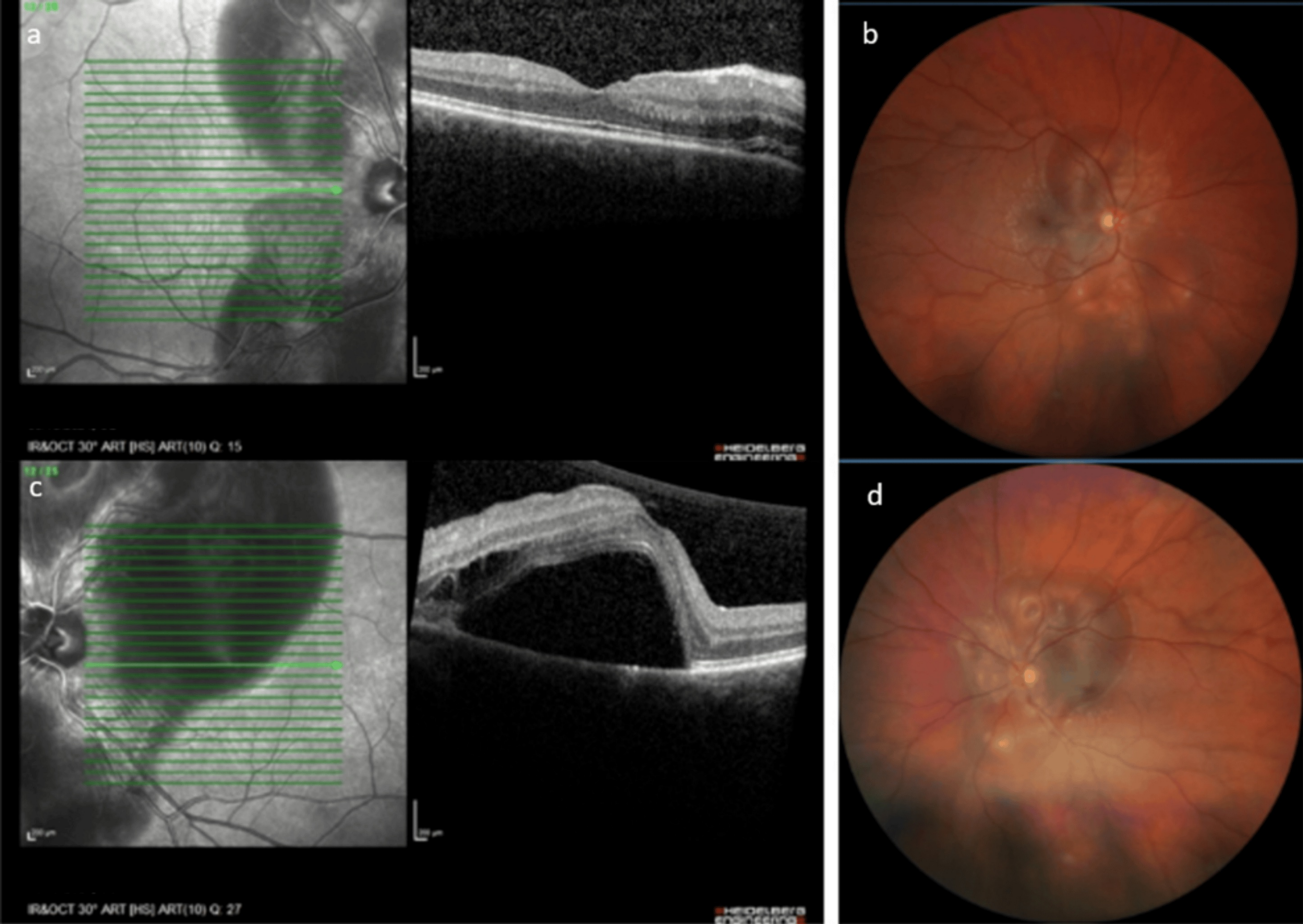
Thirty two weeks of pregnancy 1a. Macular OCT of OD demonstrating a thin layer of fluid in the interpapillomacular area with no involvement of the foveola. 1b. Fundus photography of OD demonstrating a peripapillary SRD sparing the foveola. 1c. Macular OCT of OS demonstrating a large foveolar SRD. 1d. Fundus photography of OS demonstrating a peripapillary SRD involving the foveola. OCT: optical coherence tomography; OD: right eye; SRD: serous retinal detachment; OS: left eye

During her hospital stay, at 32 weeks, the patient reported decreased visual acuity (VA) in the left eye. Ophthalmological examination found a best-corrected visual acuity (BCVA) of 20/20 in the right eye (OD) and 20/32 in the left eye (OS). Slit-lamp examination was normal in both eyes with no sign of inflammation. Fundus examination and optical coherence tomography (OCT, Heidelberg Engineering's OCT Spectralis; Heidelberg, Germany) demonstrated a large peripapillary SRD sparing the foveola in OD (Figures 1a, 1b), and a peripapillary SRD involving the foveola in OS (Figures 1c, 1d), explaining the decrease in VA. The highest blood pressure recorded at that time was 134/82 mmHg. The maximum recorded blood pressure during hospitalization was 138/94 mmHg, which was not deemed high enough according to institutional obstetric protocols to initiate antihypertensive treatment.

The ophthalmological findings were communicated to the gynecologists. Given signs of preeclampsia with severe features (worsening renal function and bilateral SRD) and failure of induction with a balloon catheter, the obstetric team performed a cesarean section at 32+3 weeks.

**Figure 2:**
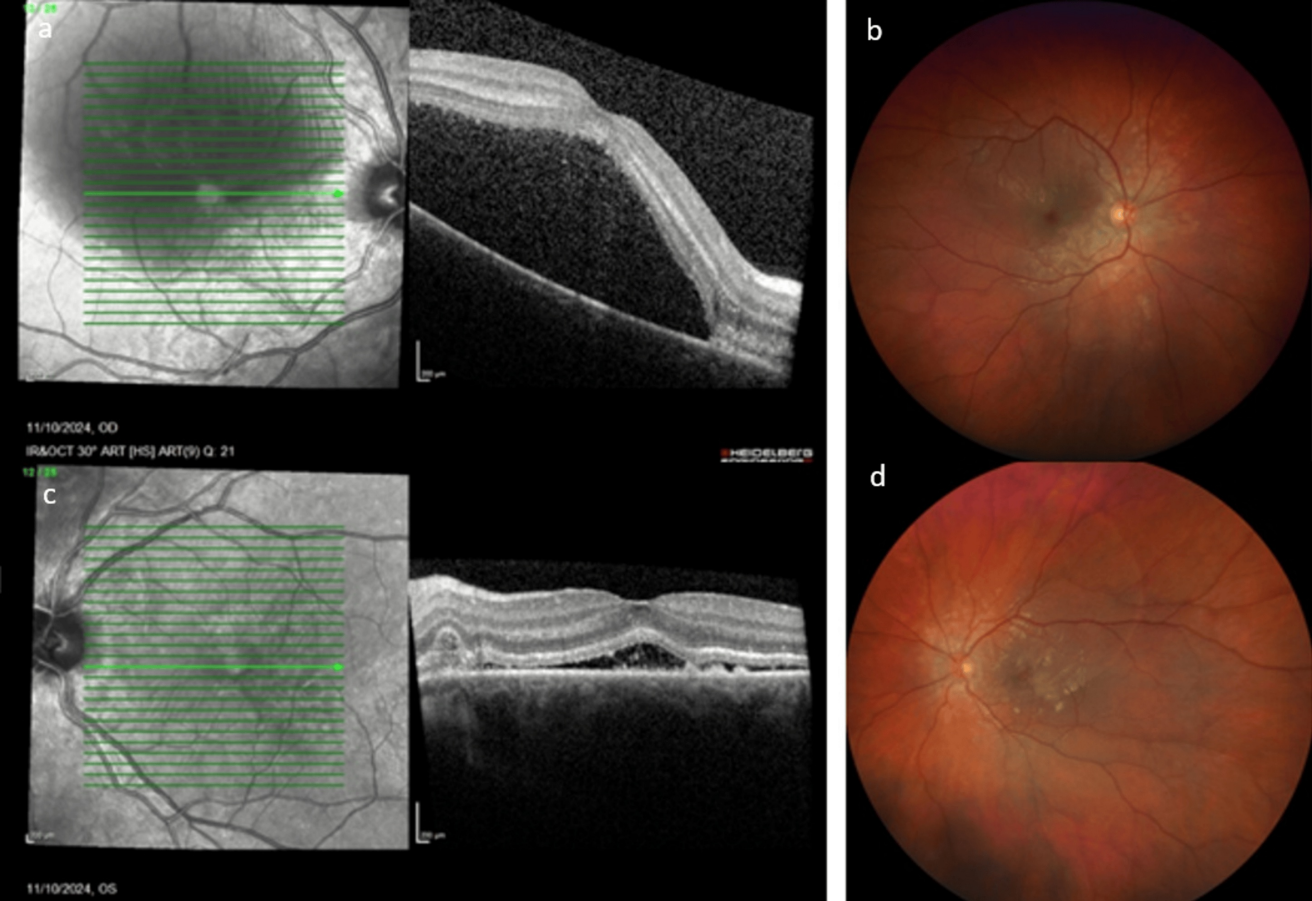
Three days post-partum 2a. Macular OCT of OD demonstrating a large foveolar SRD. 2b. Fundus photography of OD demonstrating a peripapillary SRD involving the foveola. 2c. Macular OCT demonstrating serous subretinal fluid with hyperreflective subretinal material. 2d. Fundus photography of OS demonstrating decreased peripapillary SRD involving the foveola. OCT: optical coherence tomography; OD: right eye; SRD: serous retinal detachment; OS: left eye

Based on the clinical context, the initial diagnosis was pregnancy-related SRD. As it usually resolves spontaneously with preeclampsia management, no ophthalmic treatment was initiated, and the patient was scheduled for a close follow-up. In fact, the first follow-up took place five days after the initial assessment to ensure that no new signs or symptoms had appeared; otherwise, other diagnoses would have been considered. This was three days after delivery, and the patient reported subjective improvement in OS VA and slight worsening in OD VA. Fundus examination and OCT showed progression of SRD in OD with now foveal involvement (Figures 2a, 2b). Left-eye OCT showed a reduction in subretinal fluid with hyperreflective subretinal material suggestive of fibrinous exudation (Figure 2c). The improvement of SRD was confirmed by fundus examination (Figure 2d).

**Figure 3:**
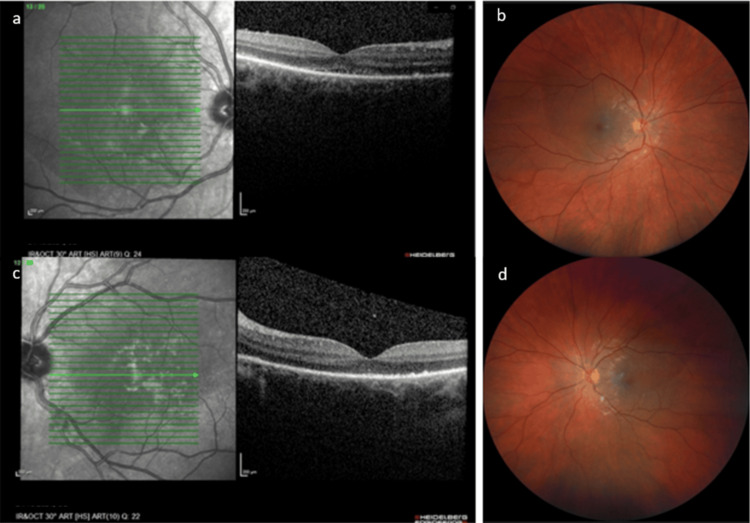
Fourteen days post-partum 3a. Macular OCT of OD demonstrating a small foveolar SRD and RPE remodeling. 3b. Fundus photography of OD. 3c. Macular OCT of OS demonstrating complete resolution of SRD and RPE remodeling. 3d. Fundus photography of OS. OCT: optical coherence tomography; OD: right eye; SRD: serous retinal detachment; RPE: retinal pigment epithelium; OS: left eye

At two weeks postpartum, the patient reported less blurred vision, but she was still experiencing visual discomfort, although BCVA was 20/20 in both eyes. Fundus examination showed a significant reduction of the SRD in OD (Figure 3a), while OCT still demonstrated a thin layer of fluid (Figure 3b). Complete resolution was observed in OS (Figures 3c, 3d). 

**Figure 4:**
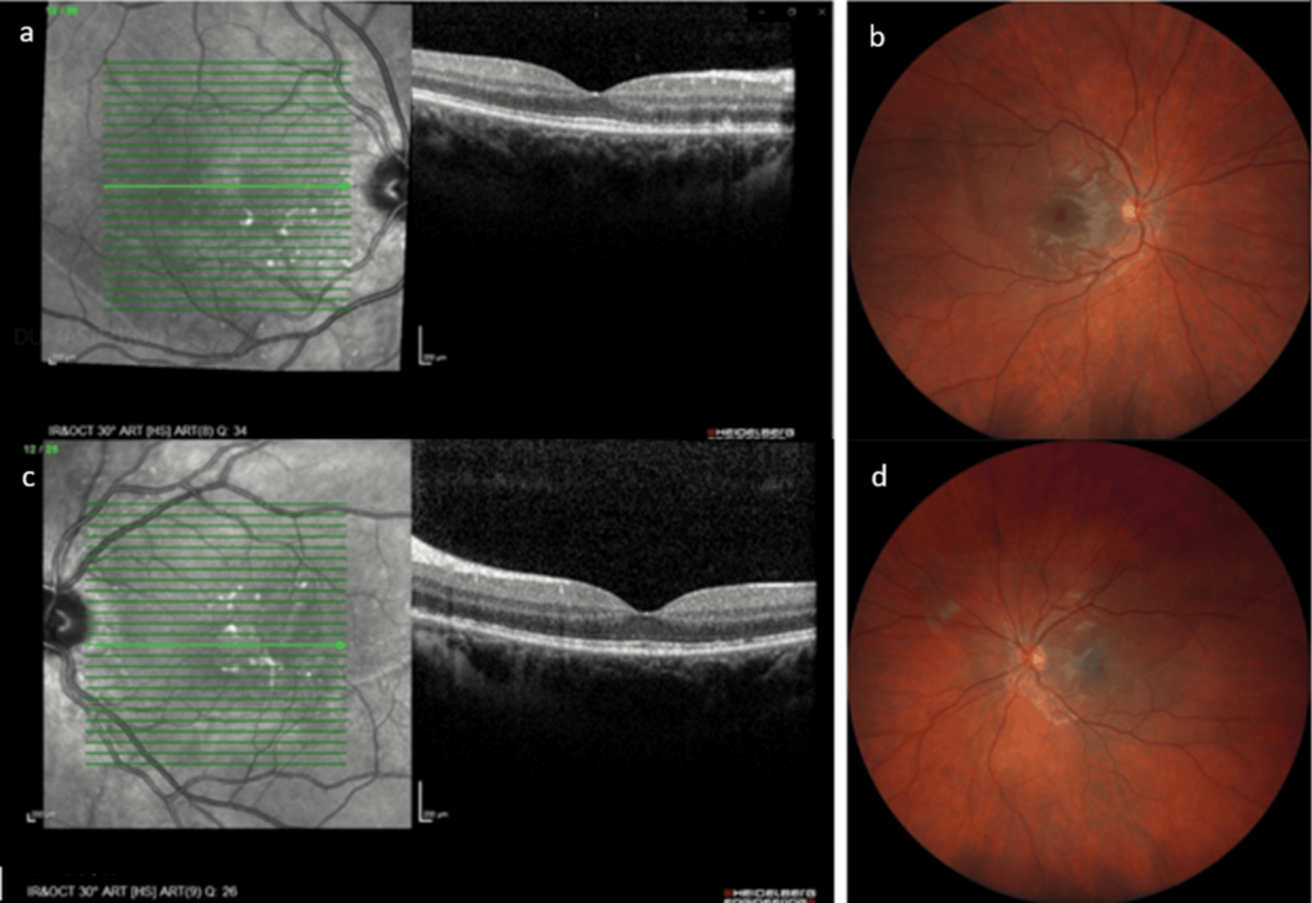
One month post-partum 4a. Macular OCT of OD demonstrating complete resolution of SRD and RPE remodeling. 4b. Fundus photography of OD. 4c. Macular OCT of OS demonstrating complete resolution of SRD and RPE remodeling. 4d. Fundus photography of OS. OCT: optical coherence tomography; OD: right eye; SRD: serous retinal detachment; RPE: retinal pigment epithelium; OS: left eye

At one month postpartum, the patient had full visual recovery (BCVA 20/20 OU). Fundus examination showed delineation of the previous SRD area (Figures 4a, 4c), but no fluid was detected by OCT. Areas of retinal pigment epithelium remodeling were observed (Figures 4b, 4d).

**Figure 5:**
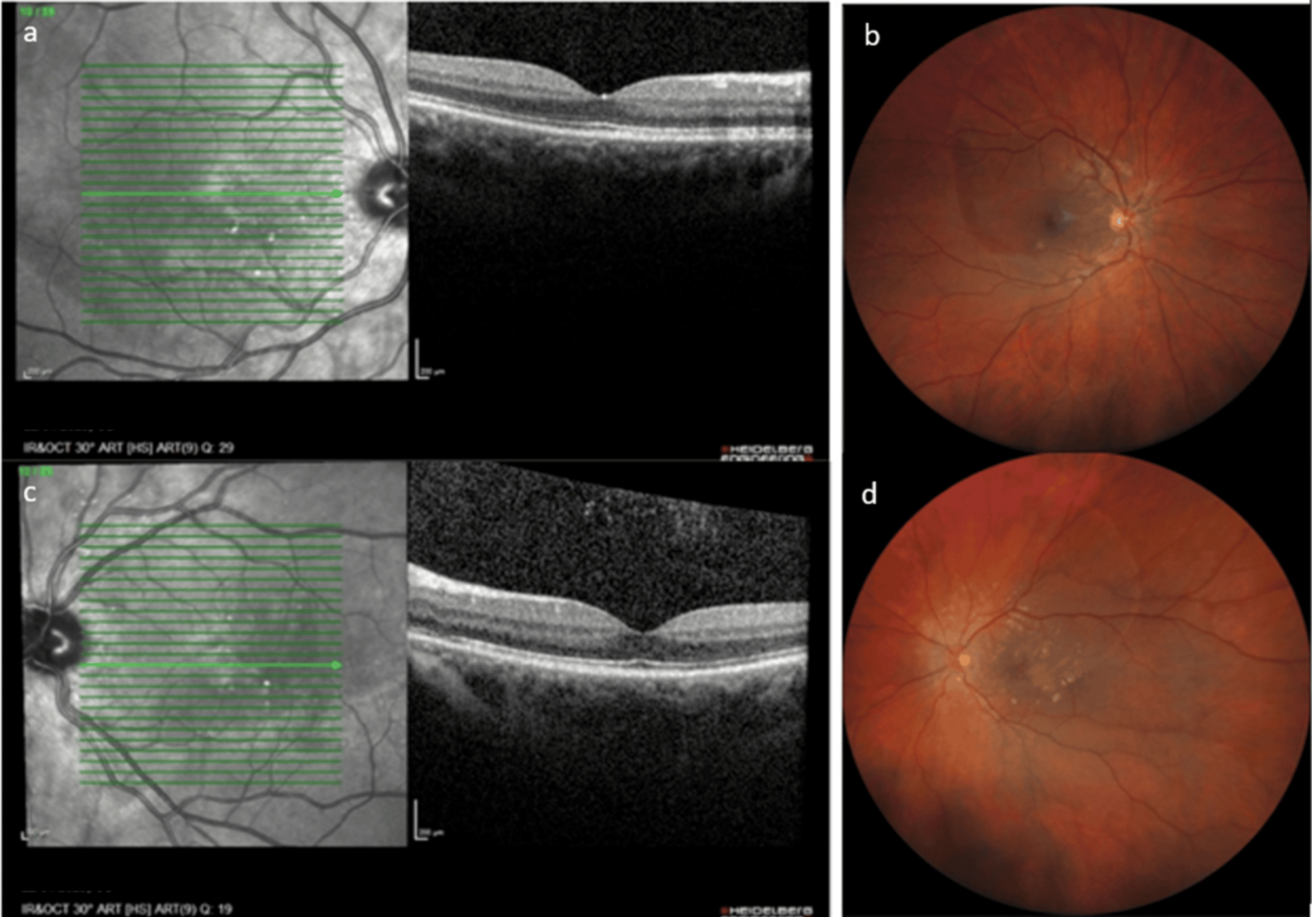
Three months post-partum 5a. Macular OCT of OD demonstrating a normal macular structure with intact retinal layers. 5b. Fundus photography of OD. 5c.Macular OCT of OS demonstrating a normal macular structure with intact retinal layers. 5d. Fundus photography of OS. OCT: optical coherence tomography; OD: right eye; SRD: serous retinal detachment; RPE: retinal pigment epithelium; OS: left eye

Progressive spontaneous resorption of SRD was observed, with complete anatomical resolution on OCT by three months postpartum (Figure 5).

## Discussion

Preeclampsia is a multisystemic disorder characterized by new-onset hypertension after 20 weeks of gestation, associated with 1 or more of the following features: proteinuria, maternal organ dysfunction, or uteroplacental dysfunction [4]. It is thought to result from placental dysfunction leading to the release of various circulating factors into the maternal blood, responsible for inflammation and endothelial dysfunction [4].

The clinical spectrum of preeclampsia includes severe preeclampsia, eclampsia, and HELLP syndrome [3]. Hypertension is defined as a blood pressure of 140/90mmHg or higher. Values ≥160/110mmHg are considered to indicate severe preeclampsia [5]. Eclampsia denotes seizures that occur in a woman with pre-eclampsia, not attributable to any other causes, while HELLP syndrome is characterized by hemolysis, elevated liver enzymes, and low platelets [3].

Various ophthalmic manifestations may appear during preeclampsia, including serous retinal detachment [2]. The latter is a rare condition associated more frequently with severe preeclampsia or HELLP syndrome. A recent review by Haikal et al. found that SRD was more commonly observed in primigravida women, frequently in association with severe features of preeclampsia or with HELLP syndrome, and was often detected in the postpartum period [6].

The pathophysiology of SRD in preeclampsia is not fully elucidated. Proposed mechanisms include choroidal circulatory dysfunction secondary to intense hypertensive choroidal vasospasm, leading to RPE dysfunction and subsequent accumulation of subretinal fluid [7,8]. Indeed, hypertension may promote the release of endogenous vasoconstrictor agents, resulting in choroidal vessel obstruction and ischemia, overlying RPE pump failure, and leak of fluid to the subretinal space [9,10]. While fluorescein and indocyanine green are generally avoided during pregnancy due to the risk of teratogenicity, postpartum angiography and multimodal imaging have demonstrated choroidal hypofluorescence (ischemia, Elschnig spots) and late-phase hyperfluorescence due to leakage at the RPE, consistent with these mechanisms [9]. In HELLP syndrome, hemolysis and microangiopathic processes may cause choroidal capillary obstruction and focal ischemia, which may partly explain the higher incidence of SRD in that subgroup [3]. As illustrated by our case report, SRD may also develop in the absence of arterial hypertension, suggesting that additional pathogenic mechanisms may be involved and highlighting the complexity and the gaps in our current understanding of its pathophysiology.

Clinically, SRD typically presents with decreased visual acuity, metamorphopsia, or a central scotoma, depending on the location and extent of the detachment. OCT is a noninvasive, sensitive tool for detecting subretinal fluid and monitoring resolution and should be reported when available. Despite the clinical context, it is important to consider the differential diagnosis of SRD, including central serous chorioretinopathy and inflammatory choroiditis. In the present case, the bilateral presentation of SRD in association with renal involvement, together with its spontaneous resolution without specific treatment, further supports the diagnosis of pregnancy-related SRD. Close follow-up was therefore important to monitor the proper progression of the condition.

Although SRD can be disabling for patients due to the decrease in visual acuity caused by the central location of the SRD, postpartum remission is the rule in most reported cases, with a maximum resolution time of 12 weeks [3,6,11-13]. Residual changes, such as RPE irregularities or ellipsoid zone disruption, have been described rarely; even when structural changes persist, functional recovery is often substantial [1]. Management is therefore conservative, with no requirement for medical or surgical ophthalmic intervention. Better blood pressure control and appropriate obstetric management, including delivery when maternal or fetal indications exist, are the cornerstones of therapy [14]. Ophthalmic interventions are rarely required. Close follow-up with visual acuity testing and OCT is recommended to document resolution.

As mentioned earlier, most reported cases of SRD occur in the setting of markedly elevated blood pressure or HELLP syndrome. Haikal et al. noted that all patients in their review had severe hypertension (≥160/110 mmHg), except for 1 patient with SRD and blood pressure below 160/90 mmHg; however, this patient later developed HELLP syndrome [6]. A unique case report published by Hussain et al. describes a case of SRD in a patient with proteinuria but without documented hypertension or HELLP syndrome [15].

Our case contributes to the literature by documenting bilateral SRD in a patient without markedly elevated blood pressure (maximum recorded blood pressure 138/94 mmHg) and without HELLP syndrome, underscoring that SRD can occur even in the absence of severe hypertension and that clinicians should maintain a low threshold for ophthalmic assessment in pregnant or postpartum patients reporting visual symptoms.

## Conclusions

Serous retinal detachment is an uncommon but potentially vision‑threatening complication of preeclampsia that may occur even in the absence of severe hypertension. Prompt ophthalmologic assessment with OCT and multidisciplinary obstetric management facilitates diagnosis and appropriate maternal-fetal decision-making. Conservative treatment with optimization of blood pressure and delivery when indicated typically results in complete recovery.
